# Portfolio Efficiency Tests with Conditioning Information—Comparing GMM and GEL Estimators

**DOI:** 10.3390/e24121705

**Published:** 2022-11-22

**Authors:** Caio Vigo-Pereira, Márcio Laurini

**Affiliations:** 1 cQuant.io and Department of Economics, University of São Paulo, Ribeirão Preto 14040-905, Brazil; 2 Department of Economics, School of Economics, Business Administration and Accounting at Ribeirão Preto (FEA-RP/USP), University of São Paulo, Av. dos Bandeirantes 3900, Ribeirão Preto 14040-905, Brazil

**Keywords:** portfolio efficiency, conditional information, efficiency tests, GEL, GMM, C12, C13, C58, G11, G12

## Abstract

We evaluate the use of generalized empirical likelihood (GEL) estimators in portfolio efficiency tests for asset pricing models in the presence of conditional information. The use of conditional information is relevant to portfolio management as it allows for checking whether asset allocations are efficiently exploiting all the information available in the market. Estimators from the GEL family present some optimal statistical properties, such as robustness to misspecifications and better properties in finite samples. Unlike generalized method of moments (GMM) estimators, the bias for GEL estimators does not increase with the number of moment conditions included, which is expected in conditional efficiency analysis. Due to these better properties in finite samples, our main hypothesis is that portfolio efficiency tests using GEL estimators may have better properties in terms of size, power, and robustness. Using Monte Carlo experiments, we show that GEL estimators have better performance in the presence of data contaminations, especially under heavy tails and outliers. Extensive empirical analyses show the properties of the estimators for different sample sizes and portfolio types for two asset pricing models.

## 1. Introduction

The efficiency of financial allocations plays a key role in empirical asset pricing frameworks, with theoretical and practical importance in financial markets. A fundamental point is to verify empirically if the allocations are efficient, conditional on the full set of available information. Approaches to constructing efficiency tests under the conditional point of view have been quickly developing, with the work of Ferson and Siegel [1] being a fundamental reference. The use of conditional information in efficiency tests has several advantages in relation to traditional tests. The first advantage is the incorporation of additional information in the definition of the tests. This allows us to verify if the allocation was efficient based on the whole set of information available and not only the information contained in the returns and a limited set of factors. This structure allows us to verify the impact of dynamic nonlinear strategies on the efficiency of the portfolio, which is not possible in the tests based on fixed-weight combinations of the tested asset returns, as discussed in Ferson and Siegel [1].

Although this conditional structure of efficiency tests has several advantages in comparison to traditional tests, it introduces some additional complications in terms of statistical inference. The incorporation of conditional information is accomplished through the use of an additional set of instruments in the estimation and testing procedures. We need estimators that allow for the incorporation of this additional information in the parametric structure of the model, which in fact corresponds to the use of additional moment conditions. Thus, we are restricted to moment estimators with the possibility of overidentification, that is, a number of moment conditions greater than the number of fixed parameters of the model. The natural candidate for this problem is the GMM estimator [2], which appears as a generalization of the method of moments method for the case of overidentification. As the GMM estimators do not impose any restrictions on the data distribution, only being based on assumptions about the moments, this method is widely used in finance. In this article, we discuss the use of generalized empirical likelihood estimators [3], which can be seen as a generalization of the GMM estimators, where we use a non-parametric estimate of the likelihood function as a weighting function for the construction of the expected value of the moment conditions.

Cochrane [4] even says that the GMM structure fits naturally for the stochastic discount factor formulation of asset pricing theories due to the easiness of the use of sample moments in place of population moments. However, the performance of these estimators and derived tests can be negatively affected under the conditions in which the conditional tests are performed.

The first difficulty is the use of a large number of instruments related to the incorporation of conditional information in the efficiency tests. An important result is that in the instrumental variables of the estimations by the two-stage and iterated GMM estimators, there is a statistical bias term that is proportional to the number of moment conditions, as shown in Newey and Smith [3]. Thus, efficiency tests based on conditional information using GMM estimators are subjected to a bias component, which grows with the number of moment conditions (conditional information) incorporated into the tests. Hence, the great advantage of conditional tests, which is the incorporation of information, is affected by the presence of this component of bias, damaging the statistical properties of these tests.

Financial data in particular stock returns are subject to several problems, such as the presence of conditional heteroscedasticity, non-Gaussian/asymmetric distributions, and even measurement error problems due to the impact of transaction costs and the trading structure itself, which is known as market microstructure noise. GMM estimators are partially adapted to these problems since, due to their semi-parametric nature, they do not need to assume a known parametric distribution, and the possibility of using robust estimators for the presence of serial correlation and heteroscedasticity in the estimation of weighting matrices makes this method less sensitive to serial dependency problems in the first two conditional moments. However, GMM estimators can be suboptimal in the presence of data contaminations such as outliers and heavy tails. The use of higher-order moment conditions makes these estimators sensitive to these effects (e.g., [5]), and thus these estimators are not robust to these problems.

This study analyzes the use of generalized empirical likelihood (GEL) estimators, proposed by Qin and Lawless [6], to circumvent the deficiencies existing in the use of the usual estimators in testing portfolio efficiency in the presence of conditional information. This class of estimators has some special characteristics that confer better statistical properties, such as robustness to outliers and heavy-tail distributions, and better finite sample properties compared to the usual methods based on least squares and the generalized method of moments. In generalized empirical likelihood and related methods, the bias does not increase as the number of moment conditions grows (e.g., [7]), which happens with the use of conditional information. Another important feature is that some estimation methods in the GEL family of estimators have better properties in terms of robustness to contaminations such as outliers, heavy tails, and other forms of incorrect specification (e.g., [5]). Generalized empirical likelihood estimators are related to information and entropy-based estimation methods, as discussed by Judge and Mittelhammer [8], and share some of the good properties of these estimators (see [5,8] for a detailed discussion on the relationship between GEL and other classes of estimators).

Our work contributes to the portfolio efficiency testing literature by proposing an econometric structure suitable for the special features introduced by the use of conditional information in the model. This inference method is not subject to the finite sample bias problem generated by the use of additional moment conditions, and by using a non-parametric estimator for the likelihood function, it is more robust to problems with the incorrect specification of the process distribution and is efficient in the class of semiparametric models (in the sense of Bickel et al. [9]). These theoretical characteristics suggest that this method is an interesting alternative to the traditional GMM method used in the construction of efficiency tests with conditional information incorporated in the form of moment conditions.

This issue is quite relevant in practical applications in terms of portfolio management since for fund managers, it is essential to verify that asset allocations are efficiently exploiting all the information available in the market, which in the context of conditional information, is made possible by the addition of moments conditional on the realization of other variables relevant to financial management, such as Treasury-bill and corporate bond yields, inflation, and growth rates in industrial production. In this way, our work contributes by analyzing the applied performance of the GEL estimator in the construction of conditional efficiency tests.

We study the robustness of the tests with the use of GMM and GEL estimators in a finite sample context. With Monte Carlo experiments, we assess the effects that data contaminations, such as outliers and the presence of heavy tails in the innovation structure, can have on the results of efficiency tests. In general, we see that GEL has better performance when heavy tails are present, whereas regarding the presence of outliers, both the GMM and GEL can have better robustness depending on the data-generating process (DGP) we use.

We show that under the null hypothesis, tests using either GEL or GMM estimators have a tendency to over-reject the hypothesis of efficiency in finite samples. We also evaluate how efficiency tests based on GEL and GMM estimations can lead to different decisions using real datasets. The results indicate that, in general, efficiency tests using GEL generate lower estimates compared to tests using the standard approach based on GMM. Moreover, for the case that most resembles the features of a finite sample size used in finance, we see that the results of the efficiency tests are conflicting among the GEL and GMM methodologies. All these results indicate that efficiency tests based on estimators from the GEL class perform differently compared to those of GMM, especially under small samples.

Table 1 presents an overview of recent studies grouped into broad topics on how empirical likelihood and other proposed related methods have been employed in the financial economics literature. Empirical likelihood methods have been incorporated into this field over time, and a few papers explored this family of estimators focusing on this audience [10,11]. This family of estimators was employed in applications in specific contexts in asset pricing, such as for valuing risk and option pricing [12,13,14,15,16], and specifically in portfolio theory [17,18,19]. On the other hand, to address some of the issues present in the standard methods of estimation in the portfolio theory literature, Bayesian approaches were also introduced [20,21]. Alternatively, other studies focused on the statistical tests used in portfolio theory [22,23,24,25,26].

The structure of this paper is as follows. The next section introduces the methodology, presenting the asset pricing theory and the econometric models for portfolio efficiency tests for the GMM and GEL estimation methods, with an emphasis on the latter. Section 3 provides an overview of the data used. Section 4 provides the simulation experiments to evaluate the robustness of the tests under both methods of estimation. Section 5 presents the empirical results. Finally, Section 6 concludes the paper.

## 2. Methodology

### 2.1. Incorporating Conditional Information

When testing portfolio efficiency with the use of conditional information, one should seek to maximize the unconditional mean relative to the unconditional variance, where the portfolio composition strategies are functions of the information matrix. This is the approach followed by the *unconditional mean-variance efficiency* with respect to the information. It is important to compare this framework with *conditional efficiency*, where the efficiency of the mean-variance structure is evaluated under conditional means and variances. Note that under the *unconditional mean-variance efficiency* with respect to the information, the conditional information is used in the construction of the portfolio and then the efficiency is assessed unconditionally.

Start with the fundamental valuation equation,

(1) $$E[m_{t+1}R_{t+1}-1]=0$$

where $m_{t+1}$ is the stochastic discount factor (SDF), and $R_{t+1}$ is the gross return of an asset at time t+1. Assuming that there exists a subset of observable variables ${\tilde{Z}}_{t}$ from a set $Z_{t}$ of the available information at *t*, such that ${\tilde{Z}}_{t}\subset Z_{t}$, and multiplying both sides by the elements of ${\tilde{Z}}_{t}$, we obtain the *managed portfolios* approach. If the instrument $z_{t}\in {\tilde{Z}}_{t}$ is added in the pricing equation as a product, this approach is also known as the *multiplicative approach*, being the product $R_{t+1}⊗{\tilde{Z}}_{t}$ denominated *scaled returns*):

(2) $$E[m_{t+1}\left(R_{t+1}⊗{\tilde{Z}}_{t}\right)-\left(1⊗{\tilde{Z}}_{t}\right)]=0\;.$$

Intuitively, as [1] pointed out, Equation (2) asks the SDF to price the dynamic strategy payoffs on average, which may also be understood in an unconditional form. Notice that with *managed portfolios*, it is possible to incorporate conditional information and still work with unconditional moments. The main advantages of this structure are that (i) there is no need to explicitly model the conditional distributions and (ii) it avoids the range problem of the conditional information under assumption. If it was necessary to incorporate conditional information with the use of conditional moments, from (i), it would be necessary to formulate parametric models taking the risk of incorrectly defining them, whereas from (ii), it would be necessary to assume that all investors use the same set ${\tilde{Z}}_{t}$ of instruments that is included in the conditional model, which clearly incorporates a high degree of uncertainty.

The use of the generalized method of moments (GMM) is the predominant approach for estimating asset pricing models. This happens primarily because with the GMM, there is no need to impose any distributions regarding the data, requiring only assumptions about the population moment conditions. In addition, for the multiplicative approach, its structure entails that the number of instruments must exceed the moment conditions, justifying the use of the GMM. Notice that in order to make use of the GMM, all variables that comprise the moment conditions must be jointly stationary and ergodic in addition to having finite fourth moments. Thus, the sample moments $g_{T}$ of the *managed portfolios* approach can be defined as

(3) $$g_{T}=\frac{1}{T}\sum_{t=1}^{T}\left[m_{t+1}\left(R_{t+1}⊗{\tilde{Z}}_{t}\right)-\left(1⊗{\tilde{Z}}_{t}\right)\right]\;.$$

Denoting θ as the vector of the parameters to be estimated, the GMM estimator can be defined as

(4) $${\hat{\theta }}_{T}\left(\hat{W}\right)\equiv arg\min_{\hat{\theta }}g_{T}{\left(\hat{\theta }\right)}^{\prime }{\hat{W}}_{T}g_{T}\left(\hat{\theta }\right)\;,$$

where $\hat{W}$ is the conventional positive weighting matrix q×q for *q* moment conditions from the GMM estimation.

#### Empirical Likelihood Estimation

Smith [27], Owen [28], and Qin and Lawless [6] introduced a family of estimators known as *generalized empirical likelihood* (GEL). Similar to the GMM, this class of estimators can be expressed in the form of moment conditions. According to [5], GEL is a non-parametric method with the important advantage of optimal asymptotic and finite sample properties, allowing more powerful tests, more efficient estimation of the density and distribution functions, and better bootstrap methods.

Even though GEL and GMM estimators have identical asymptotic properties, in finite samples they exhibit different behaviors. As [3] discussed, a precise examination should focus on an analysis of the higher-order asymptotic bias expressions. The authors derived this higher-order asymptotic bias for the i.i.d. case and concluded that GEL estimation is preferable to the GMM because GEL has one fewer term in its second-order asymptotic bias expression. Moreover, they also demonstrated a practical implication when there is a considerable quantity of instruments. Under this situation, it is not recommended to select many instruments for a GMM estimation to avoid inflating the bias. Anatolyev [7] reached similar conclusions when comparing the second-order asymptotic bias for GEL and GMM estimators in time-series models. In summary, compared to the GMM, estimations based on GEL imply that the bias should not increase as the number of moment conditions grows.

Following [5], consider a system of restrictions on unconditional moments such as

(5) $$E[g\left(w,\theta_{0}\right)]=0\;.$$

where θ∈Θ is a k×1 vector of the true parameters, *w* is a vector of observables, ${\left\{w_{i}\right\}}_{i=1}^{n}$ is a random sample, and g(w,θ) is a vector q×1 of the moment conditions. Let $p=(p_{1},p_{2},\ldots ,p_{n})$ be a collection of probability weights assigned to each sample observation. Thus, we have the following *empirical likelihood* problem:

(6) $$\begin{array}{cc} \max_{p,\theta } & \frac{1}{n}\sum_{i=1}^{n}log\left(p_{i}\right)\\ \; & \\ \mathrm{subject}\;\mathrm{to}\; & \sum_{i=1}^{n}p_{i}g\left(w_{i},\theta \right)=0\\ \; & \sum_{i=1}^{n}p_{i}=1\;. \end{array}$$

Succinctly, we can say that estimations by GEL seek to minimize the distance between the vector of probabilities *p* and the empirical density 1/n in Equation (6). From this constraint maximization, we obtain the *saddlepoint problem*, which is given by

(7) $$\max_{\theta \in \Theta }\min_{\lambda }\frac{1}{n}\sum_{i=1}^{n}-log\left(1+\lambda^{\prime }g\left(w_{i},\theta \right)\right)\;.$$

From the solution of this problem, it is possible to obtain the empirical likelihood estimator $\hat{\theta }$ (as well as the *GEL multipliers* $\hat{\lambda }$). If the substitution is made in the *saddlepoint problem* in Equation (7) by an arbitrary criterion that is subject to certain shape conditions, one can obtain the *GEL estimator*. To do so, let ρ(υ) be a strictly concave smooth function, which satisfies ρ(0)=0, $\partial \rho \left(0\right)/\partial \upsilon =\partial^{2}\rho \left(0\right)/\partial \upsilon^{2}=-1$. Thus, the GEL estimator is given by $\hat{\theta }$ and the GEL multipliers by $\hat{\lambda }$, which are the solution of the *saddlepoint problem* below:

(8) $$\min_{\theta \in \Theta }\underset{\lambda \in \Lambda_{n}}{\sup}\sum_{i=1}^{n}\rho \left(\lambda^{\prime }g\left(w_{i},\theta \right)\right)\;,$$

where $\Lambda_{n}=\left\{\lambda :\lambda^{\prime }m\left(w_{i},\theta \right)\in \Upsilon ,i=1,\ldots ,n\right\}$ and Υ is some open set containing zero [3].

GEL moment conditions can be modified to incorporate serially correlated data. This approach is known as *smoothed generalized empirical likelihood (SGEL)* [27,29,30,31]. Let ${\left\{w_{t}\right\}}_{t=1}^{n}$ be a strictly stationary and ergodic time series. The smoothed moment conditions can be written as

(9) $$g_{t}^{w}\left(\theta \right)=\sum_{j=t-n}^{t-1}\omega \left(j\right)\left(g\left(w,\theta_{0}\right)\right)\;,$$

and the system of weights is given by $\sum_{j=-\infty }^{\infty }\omega \left(j\right)=1$ and $\omega \left(j\right)=\frac{1}{b}K\left(j/b\right)$, where K(u):(−∞,∞)→R is a symmetric, continuously differentiable kernel function, with K(0)≠0 and ∫K(u)du=1, and *b* is a bandwidth. Replacing the moment $g(w,\theta_{0})$ in the *saddle point problem* in (8) with our smoothed moment $g_{t}^{w}\left(\theta \right)$ given in Equation (9), we can obtain the ${\hat{\theta }}_{SGEL}$ estimator.

Each of the estimators that are within the GEL class uses different metrics to measure the distance. Owen [28] defined *empirical likelihood* (EL) as ρ(υ)=ln(1−υ). Kitamura and Stutzer [30] developed the estimator *exponential tilting* (ET), where ρ(υ)=−exp(υ). Finally, we have the *continuously updated estimator* (CUE), where ρ(υ) is a quadratic function. The CUE was developed by Hansen et al. [32], but it was Newey and Smith [3] who showed that this estimator can also be classified in the GEL family.

### 2.2. Tests of Efficiency

Let $f_{t}$ be a vector with dimension K×1 for the factors that comprise an asset pricing model and assume that from now on, we are working with excess returns. For a system with *N* assets, we have the following statistical structure for these models:

(10) $$\begin{array}{c} R_{t}=\alpha +\beta f_{t}+\varepsilon_{t}\\ E[\varepsilon_{t}]=0\\ E[\varepsilon \varepsilon_{t}^{\prime }]=\Sigma \\ Cov[f_{t},\varepsilon_{t}^{\prime }]=0\;, \end{array}$$

where $R_{t}$, α e $\varepsilon_{t}$ have N×1 dimensions, whereas $f_{t}$ has K×1 dimensions and β is an N×K matrix. The theoretical framework for these asset pricing models implies that the vector α=0. Therefore, the portfolio defined by *K* factors derived from a linear pricing model is said to be efficient only when the *N* estimated intercepts are not jointly statistically significant. The test of efficiency to assess whether all pricing errors are jointly equal to zero can be carried out using a Wald test, where the null and alternative hypotheses are given by

(11) $$\begin{array}{c} H_{0}:\alpha =0\\ H_{A}:\alpha \neq 0\;. \end{array}$$

whereas the test statistic is given by

(12) $$J_{Wald}={\hat{\alpha }}^{\prime }{\left[\mathrm{Cov}\left(\hat{\alpha }\right)\right]}^{-1}\hat{\alpha }\;,$$

so that under the null hypothesis, $J_{Wald}$ must have a distribution of $\chi^{2}$ with *N* degrees of freedom. However, one should remember the limitation of the Wald test that underlies the large sample distribution theory. According to [4], the test remains valid asymptotically even if the factor is stochastic and the covariance matrix of the disturbances Σ is estimated. If, on the one hand, there is no need to assume that the errors are normally distributed, on the other hand, this test ignores the sources of variation in finite samples. From the central limit theorem, the test is based primarily on the fact that $\hat{\alpha }$ has a normal distribution.

Gibbons et al. [33] derived the finite sample distribution of the null hypothesis in which the alphas are jointly equal to zero. In contrast to the $J_{Wald}$ test, this test, denoted as GRS, recognizes sample variations in the estimated covariance matrix of the disturbances $\hat{\Sigma }$. However, the test requires the errors to be normally distributed, homoskedastic, and uncorrelated. This test is defined by

(13) $$J_{GRS}=\frac{T-N-K}{N}{\left(1+E_{T}{\left(f\right)}^{\prime }{\hat{\Omega }}^{-1}E_{T}\left(f\right)\right)}^{-1}{\hat{\alpha }}^{\prime }{\hat{\Sigma }}^{-1}\hat{\alpha }\;,$$

where $E_{T}\left(\;\cdot \;\right)$ is the sample mean, and

(14) $$\begin{array}{c} \hat{\Omega }=\frac{1}{T}\sum_{t=1}^{T}\left[f_{t}-E_{T}\left(f\right)\right]{\left[f_{t}-E_{T}\left(f\right)\right]}^{\prime }\\ \hat{\Sigma }=\frac{1}{T}\sum_{t=1}^{T}{\hat{\varepsilon }}_{t}{\hat{\varepsilon }}_{t}^{\prime }\;. \end{array}$$

Therefore, under i.i.d. and normally distributed errors, the statistic test $J_{GRS}$ has an unconditional distribution as an *F* with *N* degrees of freedom in the numerator, and T−N−K degrees of freedom in the denominator. Note that by assuming $\varepsilon_{t}∼N.I.D.$, one can show that $\hat{\alpha }$ has a normal distribution and $\hat{\Sigma }$ has a Wishart distribution. Precisely,

(15) $$\begin{array}{c} \hat{\alpha }∼N\left(\alpha ,\frac{1}{T}\left[1+E_{T}{\left(f\right)}^{\prime }{\hat{\Omega }}^{-1}E_{T}\left(f\right)\right]\Sigma \right)\\ T\hat{\Sigma }∼W_{N}\left(T-2,\Sigma \right)\;, \end{array}$$

which, being the Wishart distribution a multivariate $\chi^{2}$, implies that ${\hat{\alpha }}^{\prime }{\left[\mathrm{Cov}\left(\hat{\alpha }\right)\right]}^{-1}\hat{\alpha }$ should result in an *F* distribution.

## 3. Data

The data employed can be grouped into different instruments, factors, and portfolios. The common maximum time span for all our data is 720 months (60 years) prior to December 2014. As for the factors, we used a set of five standard instruments commonly employed in this type of analysis to measure the state of the economy and form our set of conditional information. One could say that the chosen lagged variables are part of a somewhat standard set of instruments for this purpose. The first was the lagged value of the 3-month Treasury-bill yield [34]. The second was the spread between corporate bond yields with different ratings. This spread was derived from the difference between the Moody’s Baa and Aaa corporate bond yields [1,35]. Another instrument was the spread between the 10-year and 1-year Treasury-bill yields with constant maturity [1,36]. Following [34], we included the percentage change in U.S. inflation measured by the *Consumer Price Index* (CPI). Lastly, the monthly growth rate of seasonally adjusted industrial production was also used, measured by the *Industrial Production Index* [34]. All data were extracted from the historical time series provided by the Federal Reserve.

Given that we focused on the CAPM and Fama–French three-factor model, we extracted the factors for both approaches from the Kenneth R. French website (http://mba.tuck.dartmouth.edu/pages/faculty/ken.french/data_library.html accessed on 11 November 2022). The market portfolio consists of the weighted return of the value of all companies listed on the NYSE, AMEX, and NASDAQ. More precisely, the market portfolio consists of the value-weight returns of all CRSP firms incorporated in the US and listed on the NYSE, AMEX, or NASDAQ that has a CRSP share code of 10 or 11 at the beginning of month *t*, good shares and price data at the beginning of *t*, and good return data for *t*. The SMB and HML factors are computed in accordance with [37]. The first factor is the average return of three smaller portfolios subtracted from the average return of the three largest portfolios, whereas the second one is the average return of the two portfolios with high book-to-market subtracted from the average return of the two portfolios with low book-to-market.

**Figure 1:**
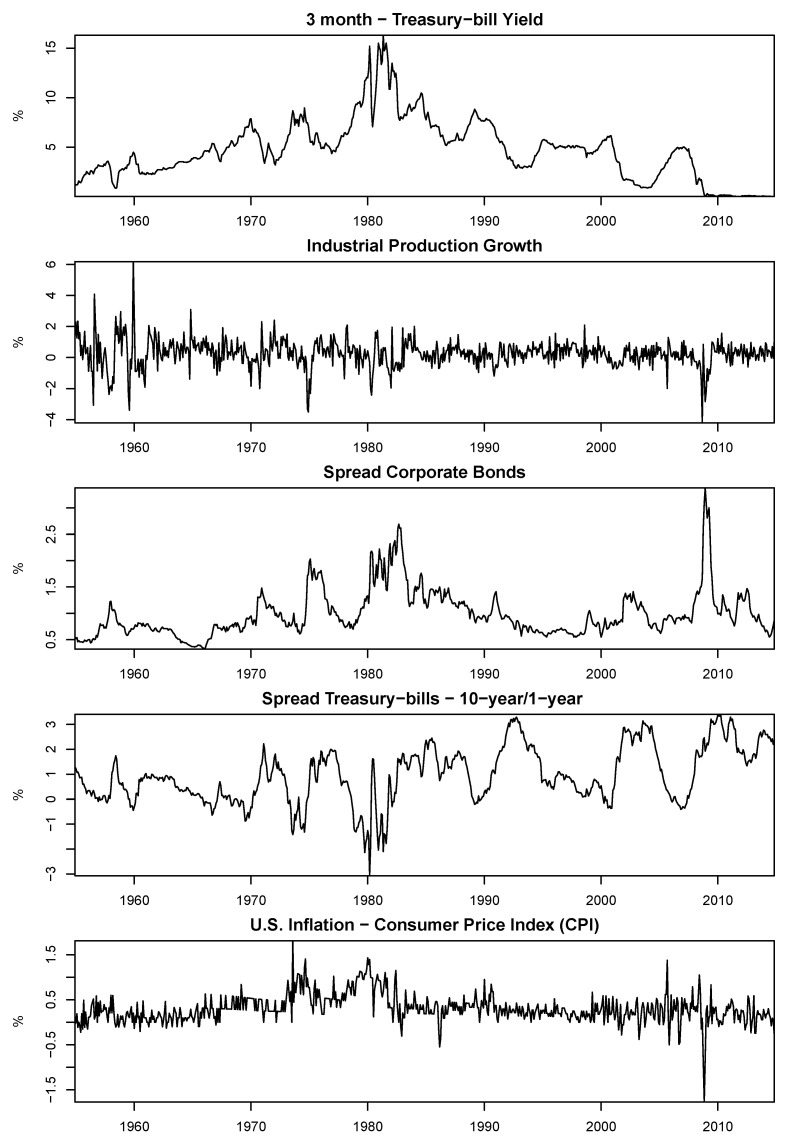
Historical series of the instruments for 720 months from January-1955 to December-2014.

**Figure 2:**
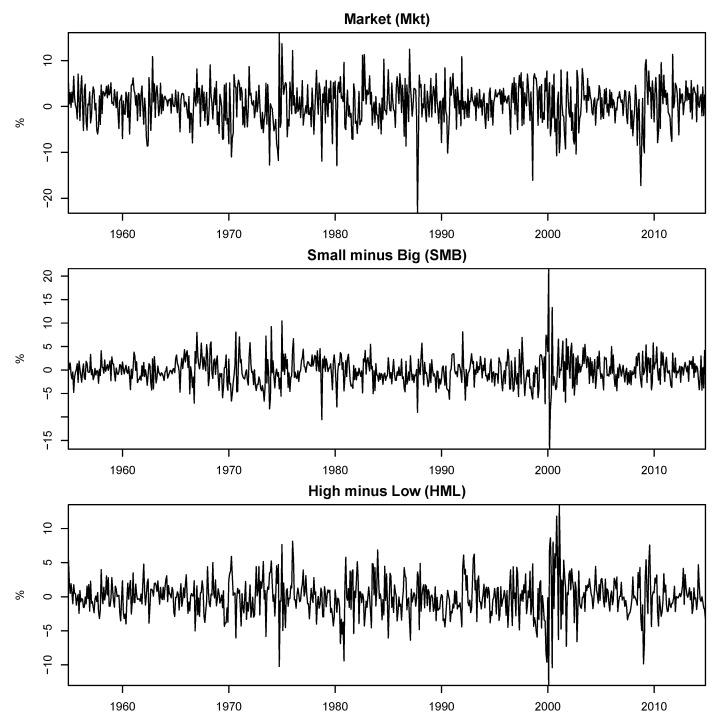
Historical series of the factors for 720 months from January-1955 to December-2014.

Figure 1 and Figure 2, respectively, present the complete historical series of the lagged state variables and factors used. From the plots of the five instruments, important events in the 60-year range of our data can be easily seen through the peaks and valleys. The oil crisis and the 2008 Great Recession are examples of events that impacted the lagged variables of the economy. Table 2 shows some descriptive statistics for the instruments and factors for the 720-month period. The first-order autocorrelation shows that the instruments were highly persistent, whereas this was not observed for the factors. Note that for most of the five instruments, the first-order autocorrelation was 97% or higher. The only instrument that could not be considered persistent was the *Industrial Production Index*, which had a first-order autocorrelation of 37%. The three factors had first-order autocorrelations lower than 20%.

We made use of the six portfolios selected with equal weights by size and book-to-market (*6 Portfolios Formed on Size and Book-to-Market (2 × 3)*). The six portfolios are constructed at the end of each June as the intersections of two portfolios based on market equity and three portfolios based on the book-equity to market-equity ratio, and include all NYSE, AMEX, and NASDAQ stocks, with market equity and positive book data regularly reported by Kenneth R. French (see http://mba.tuck.dartmouth.edu/pages/faculty/ken.french/Data_Library/six_portfolios.html for further details—accessed on 11 November 2022). Table 2 also shows the descriptive statistics of the monthly returns of these six portfolios for the same sample period. The lagged variables were used to compute the $R^{2}$ statistic. Note that the mean ranged from 0.5% to 1.2% and the standard deviation from 4.7% to 7.2%. The table also presents the first-order autocorrelations, which were generally low and between 12% and 26%, as well as the $R^{2}$ from the regressions of the five instruments on the returns. Note that the adjustment coefficient was very low for all six assets, being of the order of 2%.

## 4. Evaluating Robustness with Monte Carlo Simulations

In order to evaluate robustness, we assessed the statistical properties of the efficiency test statistics using GMM and GEL estimators in a finite sample context. The main goal here was to analyze the size of the Wald and GRS tests under different specifications. The robustness properties were of special interest since contaminations such as heavy tails and outliers may be present in this type of data. Specifically, we were interested in assessing their robustness under (i) finite samples; (ii) data contaminations, such as the presence of outliers and heavy tails in the data; and (iii) increasing numbers of moment conditions.

In our Monte Carlo experiments, we restricted the DGP of the artificial returns to be efficient. This is achieved by defining our generating process to be a function of a specific number of factors with no intercepts (i.e., setting α=0). By defining different processes for the disturbance term in this DGP, we can generate data with certain features that we are interested in assessing. We constructed four different scenarios to try to incorporate some patterns seen in real financial data. Then, we analyzed the robustness of the estimators through the size properties of the tests presented in the previous section.

To build a dataset of artificial returns, we used the actual returns from the six portfolios based on size and book-to-market and the factors from the Fama–French three-factor model. Seeking to analyze the behavior of our estimators in a finite sample context, we set the sample size to T=120. We used monthly data spanning the 120 months (10 years) prior to December, 2014. We worked with *managed portfolios* to assess the impact of a higher number of moment conditions during the estimation process. A HAC covariance matrix was used for the GMM. In order to deal with serially correlated data, we used smoothed moment conditions for GEL as in Equation (9). We used the set of five instruments from Section 3 to form our set of conditional information.

For each portfolio, we ran OLS regressions of the excess returns $R_{i,t}$ on the three factors from the Fama–French model, yielding three estimated coefficients of the parameters $\beta_{i,1},\beta_{i,2},\beta_{i,3}$. Using these estimates, we built six artificial series of returns with 120 observations, each defining a process for the disturbance ${\hat{\varepsilon }}_{i,t}^{{\mathrm{Sim}}^{*}}$. In summary, our simulations shared the following common structure:

(16) $$\begin{array}{cc} R_{i,t}^{{\mathrm{Sim}}^{*}}={\hat{\beta }}_{i,1}^{OLS}Mkt_{t}+{\hat{\beta }}_{i,2}^{OLS}SMB_{t}+{\hat{\beta }}_{i,3}^{OLS}HML_{t}+{\hat{\varepsilon }}_{i,t}^{{\mathrm{Sim}}^{*}}, & \;t=1,\ldots ,120\\ & \;i=1,\ldots ,6\;. \end{array}$$

All four scenarios used this generating process, where only the disturbance term ${\hat{\varepsilon }}_{i,t}^{{\mathrm{Sim}}^{*}}$ differentiated them. We carried out 500 artificial returns dataset simulations for each of the four scenarios. We chose to run 500 simulations due to the computational burden related to the estimation of the parameters for the efficiency tests since GEL, in particular, has a high computational cost. Below, we describe the four different scenarios we considered for defining the different processes for the error term.

**Scenario 1—Gaussian Shocks:** The first scenario was our baseline. We sought to assess the efficiency tests for both estimators (GMM and GEL) in the presence of Gaussian innovations. The generating process for ${\hat{\varepsilon }}_{i,t}^{{\mathrm{Sim}}^{*}}$ is defined by

(17) $$\begin{array}{c} {\hat{\varepsilon }}_{i,t}^{{\mathrm{Sim}}^{*}}={\hat{\xi }}_{i,t}^{\mathrm{Sim}1},\;t=1,\ldots ,120\;;\;i=1,\ldots ,6 \end{array}\;{\hat{\xi }}_{i,t}^{\mathrm{Sim}1}∼N\left(0,{\hat{\sigma }}_{i}^{2\;\mathrm{OLS}}\right)\;.$$

**Scenario 2—Shocks from a t distribution:** In the second scenario, we wanted to evaluate the efficiency tests under the presence of heavy tails. As heavy tails are characterized by more extreme values in the disturbance term, an appropriate way to model them is by using innovations drawn from a t-Student distribution. We set the parameter of this distribution to have 4 degrees of freedom in order to have fatter tails. The DGP for ${\hat{\varepsilon }}_{i,t}^{{\mathrm{Sim}}^{*}}$ is given by

(18) $$\begin{array}{c} {\hat{\varepsilon }}_{i,t}^{{\mathrm{Sim}}^{*}}={\hat{\nu }}_{i,t}^{\mathrm{Sim}2},\;t=1,\ldots ,120\;;\;i=1,\ldots ,6 \end{array}\;{\hat{\nu }}_{i,t}^{\mathrm{Sim}2}∼t\left(4\right)\;.$$

**Scenario 3—Outlier on a fixed date:** The third and fourth simulation scenarios sought to evaluate the Wald and GRS tests when outliers were present in the data. In the third case, we modeled the generating process to plug a large-magnitude shock on a fixed date in our sample. Arbitrarily, we chose to add an error in the middle of the sample, i.e., when t=60. Following the structure of the previous scenarios, the beta coefficients of each asset in the portfolio were estimated by OLS, and when t=T/2=60, there was a negative shock of 5 standard deviations randomly drawn from a normal distribution, with the variance calculated using the original data. In this case, ${\hat{\varepsilon }}_{i,t}^{{\mathrm{Sim}}^{*}}$ is defined as

(19) $$\begin{array}{c} {\hat{\varepsilon }}_{i,t}^{{\mathrm{Sim}}^{*}}={𝟙}_{t=T/2}\left({\hat{\kappa }}_{i,t}^{\mathrm{Sim}3}\right),\;t=1,\ldots ,120\;;\;i=1,\ldots ,6 \end{array}\;{𝟙}_{t=T/2}\left(\kappa_{i,t}^{\mathrm{Sim}3}\right)=\left\{ \begin{array}{cc} -{\hat{\kappa }}_{i,t}^{\mathrm{Sim}3} & ,\;\mathrm{if}\;t=T/2\\ 0 & ,\;\mathrm{if}\;t\neq T/2 \end{array}\right.\;{\hat{\kappa }}_{i,t}^{\mathrm{Sim}3}∼N\left(0,5{\hat{\sigma }}_{i}^{2\;\mathrm{OLS}}\right)\;.$$

**Scenario 4—Outlier with 5% probability:** The fourth scenario took another approach to simulating outliers. We used a probability process of extreme events, arbitrarily assuming that the probability of an outlier occurring in each period was 5%. In case of success, we added an outlier with 5 standard deviations randomly drawn from a normal distribution, with the variance estimated from the original data. In this case, the DGP of ${\hat{\varepsilon }}_{i,t}^{{\mathrm{Sim}}^{*}}$ is given by

(20) $$\begin{array}{c} {\hat{\varepsilon }}_{i,t}^{{\mathrm{Sim}}^{*}}={\hat{\xi }}_{i,t}^{\mathrm{Sim}4}-{𝟙}_{{\hat{p}}_{i,t}<0.05}\left({\hat{\kappa }}_{i,t}^{\mathrm{Sim}4}\right),\;t=1,\ldots ,120\;;\;i=1,\ldots ,6 \end{array}\;{𝟙}_{{\hat{p}}_{i,t}<0.05}\left(\kappa_{i,t}^{\mathrm{Sim}4}\right)=\left\{ \begin{array}{cc} {\hat{\kappa }}_{i,t}^{\mathrm{Sim}4} & ,\;\mathrm{if}\;{\hat{p}}_{i,t}^{\mathrm{Sim}4}<0.05\\ 0 & ,\;\mathrm{if}\;{\hat{p}}_{i,t}^{\mathrm{Sim}4}\geq 0.05 \end{array}\right.\;{\hat{p}}_{i,t}^{\mathrm{Sim}4}∼\mathrm{unif}\left(0,1\right)\;{\hat{\xi }}_{i,t}^{\mathrm{Sim}4}∼N\left(0,{\hat{\sigma }}_{i}^{2\;\mathrm{OLS}}\right)\;{\hat{\kappa }}_{i,t}^{\mathrm{Sim}4}∼N\left(0,5{\hat{\sigma }}_{i}^{2\;\mathrm{OLS}}\right)\;.$$

## 5. Results

### 5.1. Sampling Distributions of the Test Statistics

To analyze the results of the Monte Carlo experiments, we used the graphical method proposed by Davidson and MacKinnon [38]. First, we assessed the *p-value plot* that reports the empirical distribution function ($\hat{F}\left(x_{i}\right)$) of the *p*-values from the Wald and GRS tests against $x_{i}$ for any point $x_{i}$ in the $\left(0,1\right)$ interval. The empirical distribution function in this case is given by

$$\begin{array}{c} \hat{F}\left(x_{i}\right)\equiv \frac{1}{N}\sum_{j=1}^{N}{𝟙}_{p_{j}\leq x_{i}} \end{array}\;{𝟙}_{p_{j}\leq x_{i}}=\{\begin{array}{cc} 1 & ,\;\mathrm{if}\;p_{j}^{*}\leq x_{i}\\ 0 & ,\;\mathrm{if}\;p_{j}^{*}>x_{i}\;, \end{array}$$

where $p_{j}^{*}$ is the *p*-value of the *J* tests, i.e., either $p_{j}^{Wald}$ or $p_{j}^{GRS}$. If the distributions of the tests $J_{Wald}$ and $J_{GRS}$ used to calculate the *p*-values $p_{j}^{*}$ are correct, then each $p_{j}^{*}$ must be distributed uniformly (0,1). This implies that the $\hat{F}\left(x_{i}\right)$ chart against $x_{i}$ should be as close as possible to a 45° line. Hence, with a *p-value plot*, it is possible to quickly evaluate statistical tests that systematically over-reject, under-reject, or reject about the right proportion of the time. Having the actual size in the vertical axis and the nominal size in the horizontal axis, for a well-behaved test for any nominal size, its *p-value plot* should always lie close to the 45° line, as the actual size of the said test should be close enough to its nominal size, with the chance of observing a small deviation equally likely (thus, close to a uniform distribution). This feature is what makes it very easy to distinguish between tests that work well and tests that work badly. Additionally, as with these plots we are presented with how a given test performs for all nominal sizes, they are particularly useful for comparing tests that systematically over- or under-reject, or a combination of both, as one can easily identify the nominal size ranges in which the deterioration of the test occurs.

For situations where the test statistics being studied behaved close to the expected behavior, i.e., with graphs being close to the 45-degree line, the authors proposed the *p-value discrepancy plot*. This chart plots $\hat{F}\left(x_{i}\right)-x_{i}$ against $x_{i}$. According to the authors, there are advantages and disadvantages to this representation. Among the advantages of this chart, it presents more information than the *p-value plot* when the statistics of the tests are well behaved. However, this information can be spurious as it is just a result of the randomness of the experiments conducted. Furthermore, there is no natural scale for the vertical axis, which could cause some difficulties in interpretation. For the *p-value discrepancy plot*, if the distribution is correct, then each $p_{j}^{*}$ must be distributed uniformly (0,1) and the graph of $\hat{F}\left(x_{i}\right)-x_{i}$ against $x_{i}$ should be near the horizontal axis.

**Figure 3:**
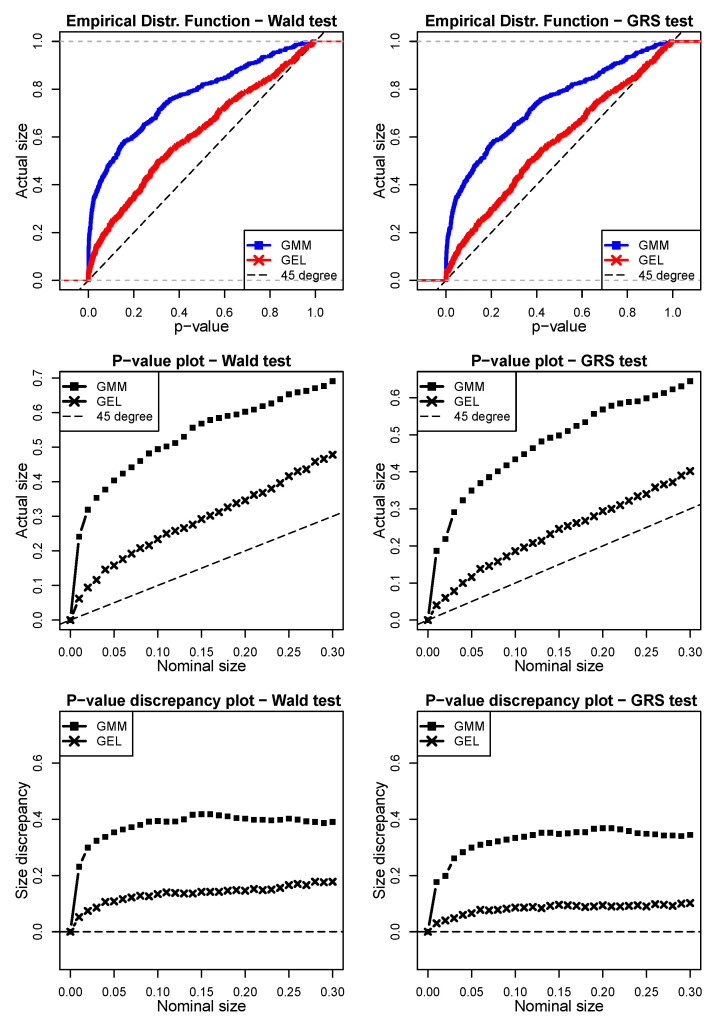
Simulation scenario 1 with Gaussian innovations (${\hat{\varepsilon }}_{i,t}^{{\mathrm{Sim}}^{*}}={\hat{\xi }}_{i,t}^{\mathrm{Sim}1}$) in Wald and GRS tests (model = Fama–French, N = 6, T = 120, 500 simulations). The left column shows the simulations for the $J_{Wald}$ test, whereas the right column shows the simulations for the $J_{GRS}$ test. The top two graphs are the empirical distribution function (EDF) of the *p*-values obtained via the GMM and GEL for both tests. The two graphs in the middle are the *p-value plots*, whereas the bottom two are the *p-value discrepancy plots*. In order to facilitate the visualization, in the EDF and *p-value plot* charts, we use dashed lines to represent the $45^{\circ }$ line. For the *p-value discrepancy plots,* the dashed lines represent the *x*-axes.

The results for the first simulated scenario derived from a Gaussian disturbance are shown in Figure 3. By analyzing the *p-value plot*, we can see that GEL provided better *p*-values than the GMM for both the Wald and GRS tests under the null hypothesis. We can see that both GEL and the GMM over-rejected for any nominal sizes. For instance, taking a 5% nominal size for the Wald test, the GMM showed an actual size (proportion of rejections under the validity of the null hypothesis) of 40.36%, whereas the size of GEL was less than half of this (15.8%). For the same 5% nominal size, the GRS test derived for the finite samples indeed performed better for both the GMM and GEL. However, GEL still had better performance. Regarding the *p-value discrepancy plot*, we can observe similar results. Based on these graphs, it is possible to observe the superiority of GEL compared to the GMM for estimating the parameters for the $J_{Wald}$ and $J_{GRS}$ tests when Gaussian shocks exist.

**Figure 4:**
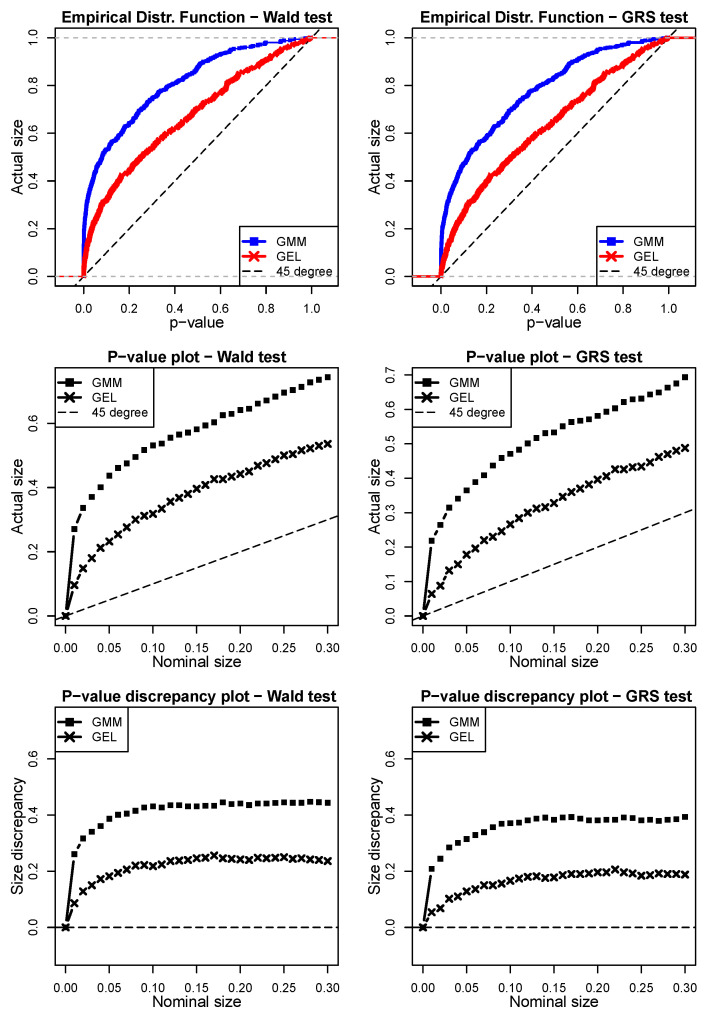
Simulation scenario 2 with shocks from a *t* distribution (${\hat{\varepsilon }}_{i,t}^{{\mathrm{Sim}}^{*}}={\hat{\nu }}_{i,t}^{\mathrm{Sim}2}$) in the Wald and GRS tests (model = Fama–French, N = 6, T = 120, 500 simulations). The left column shows the simulations for the $J_{Wald}$ test, whereas the right column shows the simulations for the $J_{GRS}$ test. The top two graphs are the EDF of the *p*-values obtained via the GMM and GEL for both tests. The two graphs in the middle are the *p-value plots*, whereas the bottom two are the *p-value discrepancy plots*. In order to facilitate the visualization, in the EDF and *p-value plot* charts, we use dashed lines to represent the 45° line. For the *p-value discrepancy plots,* the dashed lines represent the *x*-axes.

The results for the second scenario with shocks from a *t* distribution are presented in Figure 4. The structure of the graphs is the same. In this scenario, by adding a shock from a *t* distribution, we investigated the tests’ robustness for data with heavy-tail distributions. Clearly, the tests based on the GMM performed badly in the finite samples for distributions with long tails. For a 5% nominal size, the Wald test using the GMM had an actual size of 43.68%, whereas that using GEL was slightly more than half of this (23.2%). For the GRS test, the performance of both estimators improved. For the same 5% nominal size, the GMM had an actual size of 36.47 and that of GEL was 17.8. However, although one can say that the GMM performed poorly in finite samples with heavy tails compared to GEL, these results cannot hide the fact that both estimators generally over-rejected under these circumstances. Even if we consider that GEL performed better, having an actual size of nearly 5 times the 5% nominal size for the Wald test, and an actual size of more than 3 times the 5% nominal size for the GRS test, we cannot necessarily conclude that their performance was satisfactory.

**Figure 5:**
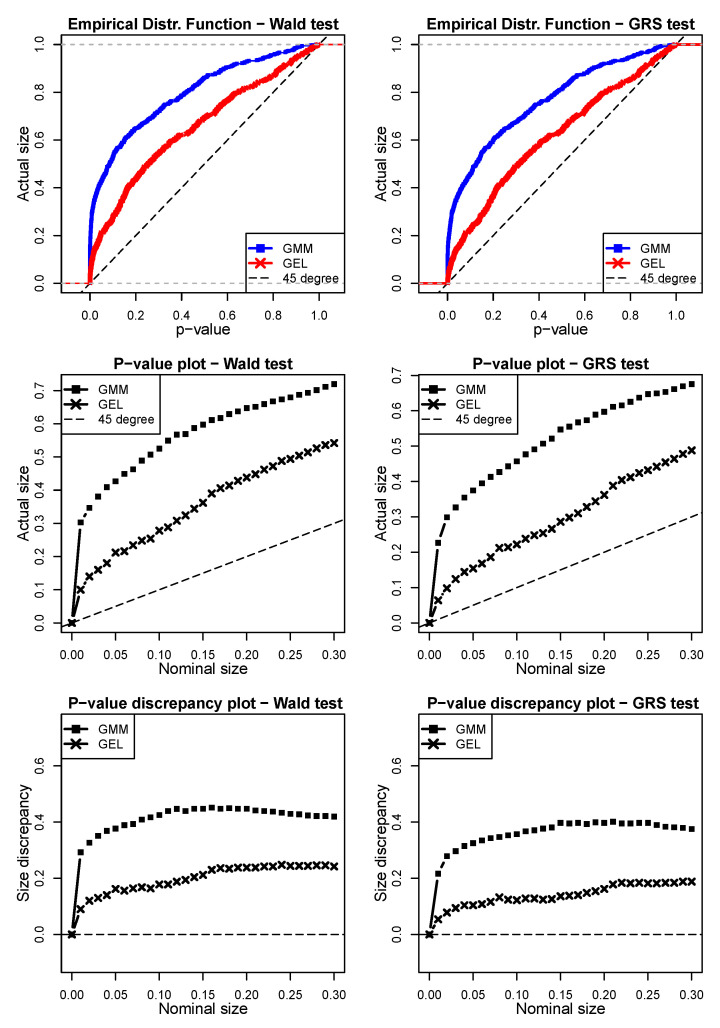
Simulation scenario 3 with shocks at t = T/2 defined by ${\hat{\varepsilon }}_{i,t}^{{\mathrm{Sim}}^{*}}={𝟙}_{t=T/2}\left({\hat{\kappa }}_{i,t}^{\mathrm{Sim}3}\right)$ in the Wald and GRS tests (model = Fama–French, N = 6, T = 120, 500 simulations). The left column shows the simulations for the $J_{Wald}$ test, whereas the right column shows the simulations for the $J_{GRS}$ test. The top two graphs are the EDF of the *p*-values obtained via the GMM and GEL for both tests. The two graphs in the middle are the *p-value plots*, whereas the bottom two are the *p-value discrepancy plots*. In order to facilitate the visualization, in the EDF and *p-value plot* charts, we use dashed lines to represent the 45° line. For the *p-value discrepancy plots,* the dashed lines represent the *x*-axes.

Figure 5 shows the results for the third scenario, with great magnitude shocks in the middle of the sample. The goal was to check robustness in the presence of outliers. Here, the evidence was similar, indicating that the GMM had worse performance than GEL under the null hypothesis. Note that both estimators always over-rejected when we added a random shock with 5 standard deviations in the middle of the sample.

**Figure 6:**
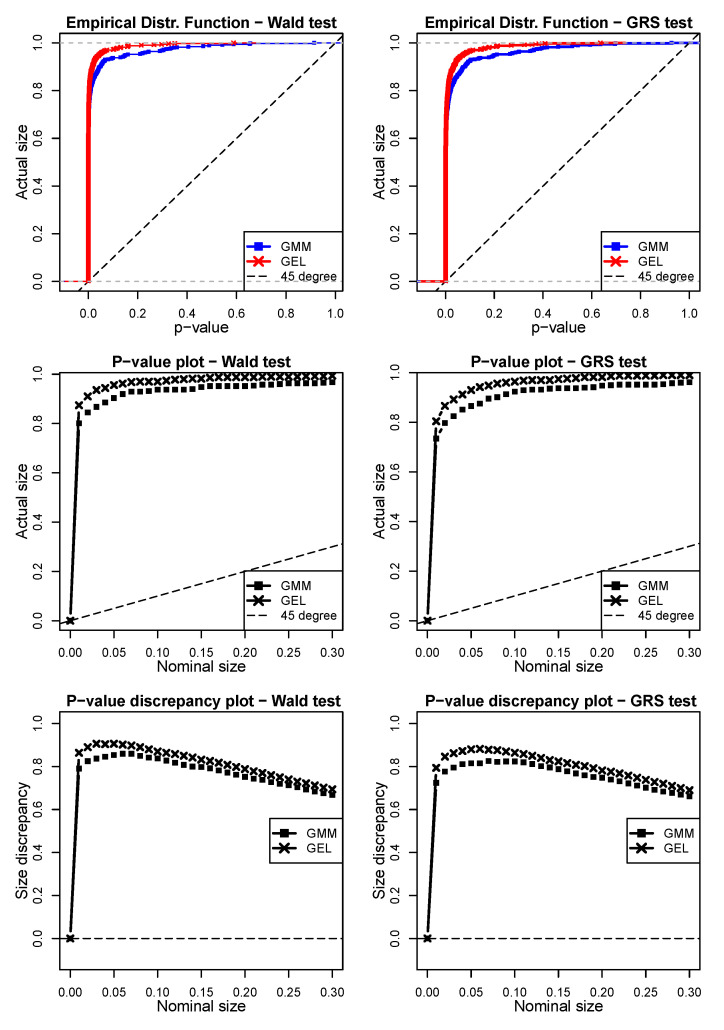
Simulation scenario 4 with shocks defined by ${\hat{\varepsilon }}_{i,t}^{{\mathrm{Sim}}^{*}}={\hat{\xi }}_{i,t}^{\mathrm{Sim}4}-{𝟙}_{{\hat{p}}_{i,t}<0.05}\left({\hat{\kappa }}_{i,t}^{\mathrm{Sim}4}\right)$ in the Wald and GRS tests (model = Fama–French, N = 6, T = 120, 500 simulations). All three left panels are the simulations for the $J_{Wald}$ test, whereas the three right panels are the simulations for the $J_{GRS}$ test. The two top panels are the EDF graphics of the *p*-values obtained via the GMM and GEL for both tests. The two central panels are the *p-value plots*, whereas the two bottom panels are the *p-value discrepancy plots*. In order to facilitate the visualization, in the EDF and *p-value plot* charts, dashed lines represent the 45° line. For the *p-value discrepancy plots,* the dashed lines represent the *x*-axes.

Finally, in Figure 6, we can see the results for the fourth scenario in which we also sought to evaluate robustness to outliers. Here, we obtained interesting results that differed from the earlier ones. The $J_{Wald}$ and $J_{GRS}$ tests based on the GMM estimations showed better results than those based on GEL for any nominal size we choose. However, note that this superiority was tenuous, being more discernible for nominal values below 10%. Taking a 5% nominal size, the Wald test with the GMM has an actual size of 90.34%, whereas that using GEL was 95.6%. For the GRS test, assuming the same 5% nominal size, the size of the GMM was 86.5% and that of GEL was 93%. By analyzing the *p-value discrepancy plots,* we can observe a similar pattern with an important feature; for both tests, both the GMM and GEL estimations tended to consistently improve performance after reaching a peak of discrepancy around a nominal size of 5%.

In summary, by analyzing all the results presented in this section, it is possible to observe that efficiency tests in finite samples with GEL estimations tend to have better performance compared to estimations via the GMM. Furthermore, tests using GEL are more robust to the presence of heavy tails. To assess the robustness for outliers, depending on the generating process assumed, both the GMM and GEL can be advantageous. However, these results also demonstrate that whatever estimator and test we evaluate, in general, the Wald and GRS tests have a tendency to over-reject.

### 5.2. Empirical Analysis

Briefly, in this section, we show how efficiency tests based on the GEL and GMM estimations can lead to different decisions using real datasets. We evaluated both methods (i) with no conditional information and (ii) when a *managed portfolios* structure was used. To do so, the analysis was conducted by comparing the test results for the different sample sizes, as well as for the two asset pricing models (CAPM and the Fama–French three-factor model), employing the efficiency tests defined according to Section 2.2. For all portfolios, testing their efficiency should be seen as testing whether the factors from each of the asset pricing models explain the portfolios’ average returns. For the CAPM, the interpretation was made by assessing whether using the individual historical returns with a unique risk factor (i.e., the *Mkt* factor) yielded an efficient portfolio (i.e., when the estimated intercepts are not jointly statistically significant), whereas for the Fama–French three-factor model, we evaluated whether the three risk factors used in Equation (11) (namely, *Mkt*, *SMB*, and *HML*) yielded a similar statistical conclusion when jointly evaluating the vector of the estimated alphas.

Table 3 presents the estimation results of the GMM and GEL when no conditional information was used in the asset pricing moments for an increasing sequence of months, starting with the last 60 months and extending the window up to 1020 months. Each sample begins in January of a given year and ends in December 2014. The table also presents the estimations of the two asset pricing models of interest for each time interval, the capital asset pricing model (CAPM) and the Fama–French (FF) three-factor model. Initially, by examining the test results using either the GMM or GEL, we noticed that for all periods over 180 months, both the CAPM and Fama–French models showed strong evidence for rejecting the hypothesis of efficiency for each model. However, for a short *T*, we observed strong disagreement between both methodologies, whereas for T=60 (i.e., 5 years), we saw no evidence for rejecting the efficiency using either the GMM or GEL for both models, and in the tests for T=90, T=120, and T=150, the GMM and GEL pointed in opposite directions.

For 90 months, the GMM rejected efficiency at a 5% significance level for the CAPM using either the Wald or GRS tests. We did not observe the same results using GEL for the same sample size. For the Fama–French model, we did not see such a strong disagreement between them. For 120 months, we saw similar results. With GEL, the *p*-values for the Wald and GRS tests were 0.30 and 0.35, respectively, for the CAPM model. With the GMM, these *p*-values were much smaller and provided evidence against the null hypothesis that the alphas were jointly equal to zero at a standard 5% significance level. For the Fama–French model, the *p*-values generated by the GMM and GEL were very similar: 0.02 and 0.05 (Wald) and 0.04 and 0.08 (GRS), respectively. For T=150 months, the same pattern was repeated. The *p*-value for the CAPM using GEL of the Wald statistic was 0.29, whereas the *p*-value of the *F* distribution under the assumption of normality given by the GRS test was 0.33. The GMM provided much smaller *p*-values, with both tests showing evidence for rejecting the efficiency hypothesis for a significance level of 5%. For the Fama–French model, the difference between the *p*-values using either GEL or the GMM was smaller. Thus, the divergence between them was more tenuous.

In Table 3, overall, we can see some evidence to endorse the simulation results presented in Section 4, as the GMM over-rejected the null hypothesis compared to tests conducted via GEL, especially in a finite sample context.

Table 4 presents the results of the efficiency tests for the *multiplicative* approach. Here, we use *managed portfolios*, where five lagged variables were used as instruments. In the Appendix A, we extend the analysis to different portfolios with higher numbers of assets (e.g., N = 25 and N = 49).

A quick inspection of the results of the tests shows us compelling evidence for rejecting the efficiency for all intervals of 180 months and above for all tests and models based on estimations from either the GMM or GEL. Although for longer periods the *p*-values were virtually zero, for T=60, T=120, and T=150 months, the inference tests using the GMM and GEL were conflicting. Singularity problems may have occurred during the estimations, impeding the inversion of the covariance matrix. These cases are shown as “NA”. For T=90, we could not perform the tests for both models using the GMM. Even though we obtained estimates for the CAPM coefficients using the GMM, we were not able to invert the covariance matrix and perform the tests. For the CAPM, GEL showed no indication to reject the efficiency (for T=120 and T=150), whereas the GMM did (*p*-values were practically zero for the Wald and GRS tests). The results are similar to the case in Table 3 where no instruments were used.

With the use of instruments, the tests of efficiency for the Fama–French model did not necessarily provide different inferences regarding the rejection of the null hypothesis. However, we still saw that the GMM generated smaller *p*-values for both tests than GEL. However, for T=60, the GMM and GEL strongly disagreed, where the GMM generated *p*-values higher than 10% and GEL had *p*-values practically equal to zero.

In order to connect these results with those from the Monte Carlo experiments performed under the different data contamination scenarios from Section 4, there are some particularities to be taken into consideration, as the results shown in Table 4 might be influenced, unlike those of the controlled Monte Carlo experiments. In fact, there is a range of complexities to be controlled in order to be able to make a fair comparison. First, embodied in our empirical results is the fact that the true DGP that generated the real data used in this analysis is unknown; we just relied on the most common factor specifications for the pair of models employed. In the case of the incompleteness of the risk factors, this inherently affects the results of any of the tests as the power and size might be impacted in distinctive ways, independently of the estimation procedure employed. Similarly, the correct test specification is fundamental (see [39] for a discussion of an alternative formulation of the GRS test). All of these issues could naturally lead to conclusions in either direction with regard to the observed rejections, given the true unknown DGP. In light of these points, the results here for the comparable cases in both analyses in which we used *managed portfolios* with a sample size of T=120 evaluated under the Fama–French model show only marginal differences (slightly higher GMM *p*-values than GEL ones). Given this magnitude of divergence in the *p*-values, one cannot argue in favor of the validation or not of the previous results solely based on these cases.

## 6. Conclusions

We evaluate the behavior of the GMM and GEL estimators in tests of portfolio efficiency. We argue that both estimators have different statistical features, and therefore, tests of portfolio efficiency based on them may reflect these differences.

First, we assess the robustness of the tests with the use of the GMM and GEL estimators in a finite sample context. Defining different DGPs to incorporate different specifications, we perform several Monte Carlo experiments to examine the effects that distortions in the data can have on tests of efficiency, and consequently, on decisions based on these results. In general, we see evidence that GEL estimators have better performance when heavy tails are present. Depending on the characteristics of the DGP chosen, both the GMM and GEL can have better robustness to outliers. However, under the null hypothesis, for both estimators, the Wald and GRS tests have a tendency to over-reject the hypothesis of efficiency in finite samples.

Using returns from real datasets in our analysis, we see that (i) in general, efficiency tests using GEL generate lower estimates (higher *p*-values) and (ii) when the sample size has finite characteristics, with low *N* and *T*, we note that the results are conflicting among the methodologies. These results may be evidence that estimators from the GEL class perform differently in small samples. In addition, they show that tests based on the GMM have a tendency to over-reject the null hypothesis of efficiency.

The results obtained in our work indicate some limitations of the use of GEL in the construction of efficiency tests, especially in empirical applications. Although the use of this method leads to improvements in properties in finite samples and greater robustness in relation to the presence of heavy tails, as discussed in Section 5.1, the GEL-based tests still show the over-rejection tendency that is also present in the tests based on GEL in the GMM. Another possible limitation is the possibility of local optima in numerical maximization procedures. As discussed in Anatolyev and Gospodinov [5], a numerical optimization with respect to the conditional structural parameters in empirical likelihood models can be hampered by the presence of local minima, possible singularities, and convergence problems due to the fact that the Hessian is not guaranteed to be positively defined during a numerical optimization. Although it is possible to use more robust optimization methods in relation to these problems, especially in empirical analysis, there is a risk of estimating a local optimum due to the dependence on the choice of initial values.

An interesting generalization of our work is the construction of portfolio efficiency tests in the presence of conditional information using other estimators related to the empirical likelihood approach. As discussed in Anatolyev and Gospodinov [5], empirical likelihood can be viewed as a member of a general family of minimum contrast estimators, especially the class of power-divergence-based estimators. By placing restrictions and some modifications on the general Cressie–Read [40] divergence function, it is possible to obtain the empirical likelihood, exponential tilting, Euclidean likelihood, GMM estimator with continuous updating, exponentially tilted empirical likelihood, and a version of the Hellinger distance estimator as particular cases. Although these classes of estimators are asymptotically equivalent, their properties in finite samples can be different, especially in relation to robustness to general forms of misspecification. In this aspect, the exponentially tilted empirical likelihood and Hellinger distance estimator classes have some theoretical robustness properties, which can be potentially relevant in the analysis of financial data.

Other possibilities for building and evaluating efficient portfolios involve the use of data envelopment analysis methods [41,42,43]. A comparison between the DEA methods and our analysis would require modifying the DEA methods to use conditional information in the form of moment conditions or instruments, which is not yet fully developed for this class of applications.

An important limitation of our work is the limited number of factors considered in our analysis, as we do not consider the impact of possible high dimensionality on the set of possible risk factors. The recent financial literature has discussed the possibility of a huge number of possible risk factors in a phenomenon known as the Zoo factor, as discussed, for example, by Harvey and Liu [44] and Feng et al. [45]. The high dimensionality in the number of possible risk factors would affect our analysis in several dimensions. The inclusion of a greater number of factors in the estimation of portfolio risk premiums would lead to a large increase in the number of moment conditions, especially in the context of the incorporation of conditional information, and the use of GEL estimators in this case would be advantageous in the sense that this method does not present the problem of bias in finite samples proportional to the number of moment conditions that impairs the performance of the GMM estimator. Note that our analysis assumes the usual estimation conditions, where the sample size is greater than the number of parameters of the conditional mean of the returns, and thus the context of a number of factors greater than the sample size would require the combination of the GMM and GEL estimators with some form of shrinkage, which has not yet been developed, to the best of our knowledge. The results of our empirical analysis also consider that the specification of the risk factors included in the model is correct, and thus the empirical results, in particular, the observed rejections, may reflect both the possible inefficiency of the portfolios in relation to the factors included and the impact on the power and size of the tests in the presence of omitted factors. A relevant development would be to adapt the portfolio efficiency tests in the presence of conditional information for the possible omission of factors in line with the methods developed by Giglio and Xiu [46] for the pricing of assets with omitted factors.

## Figures and Tables

**Table 1:** Synoptic Table.

	Method	Motivation	Findings/Contribution
**Topic: Overview of EL for Economics and Finance Audience**			
Parente and Smith [10]	EL	Overindentification models in economics and the restrictive statistical properties of GMM	Reviewed the statistical aspects of models defined by moment condition restrictions, emphasizing the contributions of the GEL class of estimators
Taniguchi et al. [11]	EL	EL flexibility and robustness against distributional assumptions for financial data	Applied EL to several financial problems to illustrate its flexibility
**Topic: General EL Applications in Finance**			
Glasserman et al. [12]	EL	To capture skewness and other features present in extreme outcomes	Developed a method for selecting and analyzing stress scenarios for financial risk assessment
Yan and Zhang [13]	(Adjusted) EL	EL robustness to distributional assumptions for the data or the estimation of the variance	Estimated confidence region for value-at-risk (VaR) and expected shortfall (ES)
Zhong et al. [14]	(Blockwise) EL	To circumvent some of the parametric assumptions on the stochastic process from the Black–Scholes model	Proposed an EL-based option pricing method
Almeida and Garcia [15]	EL	EL-type estimators’ robustness against distributional assumptions and whether they possess good statistical properties	Proposed alternative methods to measure the degree of the misspecification of asset pricing models
Camponovo et al. [16]	EL	To overcome the poor finite sample performance of the first-order asymptotic approximations	Introduced EL methods for interval estimation and hypothesis testing on volatility measures in different high-frequency data environments
**Topic: EL Applications in Portfolio Theory**			
Post and Potı [17]	EL + relative entropy	To account for incomplete information about the probability distribution due to heterogeneous beliefs, subjective distortion, and/or estimation errors, and avoid the statistical estimation and numerical inversion of the error covariance matrix	Formulated a portfolio inefficiency measure based on the divergence between the given probabilities and the nearest probabilities
Post et al. [18]	EL + stochastic dominance	To deal with the statistical estimation of the joint return distribution that affects the optimal weights, and thus leads to poor performance out of sample	Proposed a two-stage portfolio optimization method that asymptotically dominates the benchmark and optimizes the goal function in probability for a class of weakly dependent processes
Haley and McGee [19]	EL + Hellinger–Matusita distance	To deal with investors’ preferences in addition to the mean and variance as skewness or other higher-order moments such as kurtosis	Proposed new shortfall-based portfolio selection rules that are viable alternatives to existing methods, especially in terms of skewness preference
**Topic: Bayesian Methods in Portfolio Theory and Asset Pricing**			
Bauder et al. [20]	Bayesian statistics	Bayesian framework allows for the incorporation of subjective beliefs on the outcome of a future event	Estimated from a Bayesian perspective the determining parameters of the efficient frontier
Bauder et al. [21]	Bayesian statistics	To deal with parameter uncertainty in the mean-variance portfolio analysis, especially in relation to the extreme weights often seen in the sample efficient portfolio	Proposed a solution to the investors’ optimization problem by employing the posterior predictive distribution, which takes parameter uncertainty into account before the optimal portfolio choice problem is solved
**Topic: Statistical Tests in Portfolio Theory and Asset Pricing**			
Kao et al. [22]	Bayesian statistics	To overcome the issues associated with sampling errors associated with the ex-post Sharpe ratio of the test portfolio	Developed a Bayesian test of a test portfolio mean-variance efficiency
Kresta and Wang [23]	Std. Statistical Test	To address the data-snooping bias and evaluate the out-of-sample overperformance of different models of portfolio selection	Proposed an approach to verify the efficiency of the portfolio strategies by generating many random portfolios
Kopa and Post [24]	Std. Statistical Test	To deal with the limitation of an efficiency test that focuses exclusively on the efficiency classification and gives minimal information about directions for improvement if the portfolio is classified as inefficient	Developed a linear programming test to analyze whether a given investment portfolio is efficient in terms of second-order stochastic dominance
Linton et al. [25]	Std. Statistical Test	To overcome issues in previous tests related to statistical power and the ability to detect inefficient portfolios in small samples, and allow non-i.i.d. observations	Proposed a test for whether a given portfolio is efficient with respect to the stochastic dominance criterion
Berger [26]	EL	To circumvent the need for the relationship between endogenous and instrumental variables to be known	Proposed a test for the parameters of models defined by conditional moment restrictions and a model specification test

A survey of the literature on the main applications of empirical likelihood in asset pricing, portfolio efficiency tests, and the use of conditional information in the financial economics context.

**Table 2:** Descriptive statistics of the lagged variables, factors, and the portfolios’ returns for the 720 months (60 years) from January-1955 to December-2014.

	Mean	Std. Dev.	Min	Max	$\rho_{1}$	$R^{2}$
*Lagged Variables*						
3-month Treasury-Bill Yield	0.047	0.030	0.000	0.163	0.99	
Industrial Production Growth	0.002	0.009	−0.042	0.062	0.37	
Spread Corporate Bonds	0.010	0.004	0.003	0.034	0.97	
Spread Treasury Bills 10 year/1 year	0.010	0.011	−0.031	0.034	0.97	
U.S. Inflation Consumer Price Index (CPI)	0.003	0.003	−0.018	0.018	0.61	
*Factors*						
Market (Mkt)	0.005	0.044	−0.232	0.161	0.08	
Small minus Big (SMB)	−0.002	0.030	−0.169	0.216	0.06	
High minus Low (HML)	−0.000	0.027	−0.130	0.135	0.16	
*Six portfolios based on size and book-to-market (2 × 3)*						
Small 1 (Low)	0.005	0.072	−0.329	0.434	0.19	0.02
Small 2	0.010	0.056	−0.286	0.316	0.22	0.02
Small 3 (High)	0.012	0.057	−0.273	0.359	0.26	0.02
Big 1 (Low)	0.006	0.053	−0.265	0.205	0.12	0.02
Big 2	0.007	0.047	−0.242	0.218	0.13	0.02
Big 3 (High)	0.009	0.049	−0.209	0.264	0.12	0.02

Descriptive statistics of the 5 lagged variables, the 3 factors from the asset pricing models, and the monthly returns of the 6 portfolios based on size and book-to-market (2 × 3). The *ρ*_1_ column is the first-order autocorrelation. We also show the adjusted coefficients of determination *R*^2^ from the regressions of the monthly portfolios’ returns on the lagged instruments. The sample period is January 1955 through December 2014 (720 observations).

**Table 3:** Tests of portfolio efficiency using 6 portfolios formed on size and book-to-market (2 × 3) for 9 selected periods of time.

	Months	Wald Test			GRS Test			Wald Test			GRS Test	
		Statistic	*p*-Value		Statistic	*p*-Value		Statistic	*p*-Value		Statistic	*p*-Value
		**CAPM**						**FF**				
***GMM***	60	10.5	0.104		1.5	0.180		9.8	0.132		1.4	0.236
	90	14.7	0.022		2.3	0.045		12.4	0.054		1.9	0.098
	120	15.0	0.020		2.4	0.035		14.6	0.023		2.3	0.043
	150	13.7	0.033		2.2	0.048		17.6	0.007		2.8	0.015
	180	20.3	0.002		3.3	0.005		31.2	0.000		4.9	0.000
	240	28.4	0.000		4.6	0.000		64.4	0.000		10.3	0.000
	360	60.6	0.000		9.9	0.000		137.7	0.000		22.4	0.000
	480	73.0	0.000		12.0	0.000		219.3	0.000		35.9	0.000
	600	74.2	0.000		12.2	0.000		274.8	0.000		45.1	0.000
	720	78.5	0.000		13.0	0.000		285.8	0.000		47.0	0.000
	840	81.6	0.000		13.5	0.000		277.6	0.000		45.8	0.000
	960	83.2	0.000		13.8	0.000		268.5	0.000		44.3	0.000
	1020	70.2	0.000		11.6	0.000		242.7	0.000		40.1	0.000
***GEL***	60	7.5	0.279		1.1	0.374		8.3	0.218		1.2	0.335
	90	8.0	0.239		1.2	0.300		11.6	0.071		1.7	0.122
	120	7.2	0.304		1.1	0.351		12.5	0.052		1.9	0.083
	150	7.3	0.291		1.2	0.329		17.0	0.009		2.7	0.018
	180	17.3	0.008		2.8	0.013		27.1	0.000		4.3	0.000
	240	26.0	0.000		4.2	0.000		51.6	0.000		8.3	0.000
	360	54.0	0.000		8.8	0.000		109.6	0.000		17.8	0.000
	480	61.0	0.000		10.0	0.000		185.6	0.000		30.4	0.000
	600	51.5	0.000		8.5	0.000		221.6	0.000		36.4	0.000
	720	56.8	0.000		9.4	0.000		236.6	0.000		38.9	0.000
	840	56.6	0.000		9.4	0.000		227.4	0.000		37.5	0.000
	960	72.7	0.000		12.0	0.000		176.6	0.000		29.2	0.000
	1020	47.6	0.000		7.9	0.000		171.9	0.000		28.4	0.000

Tests of portfolio efficiency using 6 portfolios formed on size and book-to-market (2 × 3) for 9 selected periods of time: *T* = 60 (5 years), *T* = 90 (7.5 years), *T* = 120 (10 years), *T* = 150 (12.5 years), *T* = 180 (15 years), *T* = 240 (20 years), *T* = 360 (30 years), *T* = 480 (40 years), *T* = 600 (50 years), *T* = 720 (60 years), *T* = 840 (70 years), *T* = 960 (80 years), and *T* = 1020 (85 years). The tests are conducted based on both estimations’ methodologies, with the GMM results on the top and the GEL results on the bottom. Tests of efficiency under the CAPM are on the left, whereas tests under the Fama–French three-factor model (represented as “FF”) are on the right. This table presents the statistics and *p*-values of the Wald and GRS tests for each case.

**Table 4:** Tests of portfolio efficiency using 6 portfolios formed on size and book-to-market for *managed portfolios* for selected periods of time.

	Months	Wald Test			GRS Test			Wald Test			GRS Test	
		Statistic	*p*-Value		Statistic	*p*-Value		Statistic	*p*-Value		Statistic	*p*-Value
		**CAPM**						**FF**				
***GMM***	60	NA	NA		NA	NA		10.3	0.113		1.5	0.211
	90	NA	NA		NA	NA		NA	NA		NA	NA
	120	128.0	0.000		20.1	0.000		15.7	0.015		2.4	0.031
	150	39.3	0.000		6.2	0.000		57.2	0.000		9.0	0.000
	180	63.2	0.000		10.1	0.000		45.3	0.000		7.2	0.000
	240	32.1	0.000		5.2	0.000		97.5	0.000		15.6	0.000
	360	79.0	0.000		12.9	0.000		209.7	0.000		34.1	0.000
	480	111.5	0.000		18.3	0.000		310.4	0.000		50.8	0.000
	600	111.1	0.000		18.3	0.000		398.1	0.000		65.3	0.000
	720	102.4	0.000		16.9	0.000		380.0	0.000		62.5	0.000
***GEL***	60	45.2	0.000		6.7	0.000		65.6	0.000		9.3	0.000
	90	38.4	0.000		5.9	0.000		17.3	0.008		2.6	0.024
	120	5.6	0.466		0.9	0.509		16.3	0.012		2.5	0.025
	150	7.6	0.271		1.2	0.308		22.8	0.001		3.6	0.003
	180	32.2	0.000		5.2	0.000		46.3	0.000		7.3	0.000
	240	31.6	0.000		5.1	0.000		82.3	0.000		13.2	0.000
	360	63.3	0.000		10.3	0.000		174.9	0.000		28.4	0.000
	480	78.9	0.000		13.0	0.000		275.2	0.000		45.0	0.000
	600	72.2	0.000		11.9	0.000		241.8	0.000		39.7	0.000
	720	76.0	0.000		12.5	0.000		332.5	0.000		54.7	0.000

Tests of portfolio efficiency using 6 portfolios formed on size and book-to-market (2 × 3) for 6 selected periods of time: *T* = 60 (5 years), *T* = 90 (7.5 years), *T* = 120 (10 years), *T* = 150 (12.5 years), *T* = 180 (15 years), *T* = 240 (20 years), *T* = 360 (30 years), *T* = 480 (40 years), *T* = 600 (50 years), and *T* = 720 (60 years). The tests are evaluated using conditioning information when instruments are incorporated into the pricing equation. The lagged variables consisting of the conditioning information are the (i) 3-month Treasury-bill yield, (ii) industrial production growth, (iii) yield spreads of low-grade over high-grade corporate bonds, (iv) yield spreads of longterm over short-term Treasury bills (10 year/1 year), and (v) U.S. inflation (CPI). The tests are conducted based on both estimations’ methodologies, with the GMM results on the top and the GEL results on the bottom. Tests of efficiency under the CAPM are on the left, whereas tests under the Fama–French three-factor model (represented as “FF”) are on the right. This table presents the statistics and *p*-values of theWald and GRS tests for each case. “NA” represents situations in which singularity problems occurred, impeding the inversion of the covariance matrix. For both models, when *T* = 90, even though we obtained estimates for the coefficients through the GMM, we were not able to invert the covariance matrix and perform the tests.

**Figure A1:**
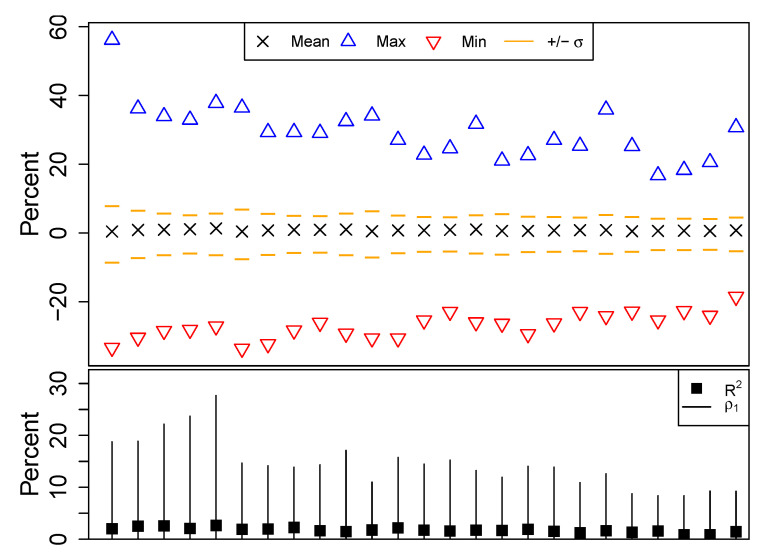
Descriptive statistics of the monthly returns for the portfolio with 25 assets for 720 months (60 years) from jan-1955 to dec-2014. The top panel shows the sample mean statistics (represented by “X”) and the max (represented by blue triangles) and the min (represented by upside-down red triangles), and the distance between the two horizontal lines represents the range of ±σ for the 720-month period. The bottom panel shows the first-order autocorrelation $\rho_{1}$ (bar) and $R^{2}$ (square points), which is the adjusted coefficient of determination as a percentage of the regression of the returns on the 5 instruments. In both panels, the *x*-axis represents the 25 assets and the *y*-axis is expressed as a percentage.

**Figure A2:**
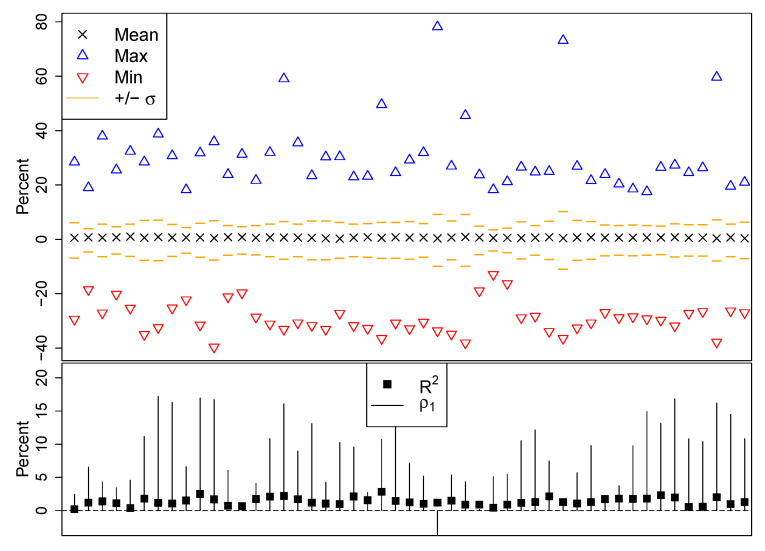
Descriptive statistics of the monthly returns for the portfolio with 49 assets for 720 months (60 years) from jan-1955 to dec-2014. The top panel shows the sample mean statistics (represented by “X”) and the max (represented by blue triangles) and the min (represented by upside-down red triangles), and the distance between the two horizontal lines represents the range of ±σ for the 720-month period. The bottom panel shows the first-order autocorrelation $\rho_{1}$ (bar) and $R^{2}$ (square points), which is the adjusted coefficient of determination as a percentage of the regression of the returns on the 5 instruments. In both panels, the *x*-axis represents the 49 assets and the *y*-axis is expressed as a percentage.

**Figure A3:**
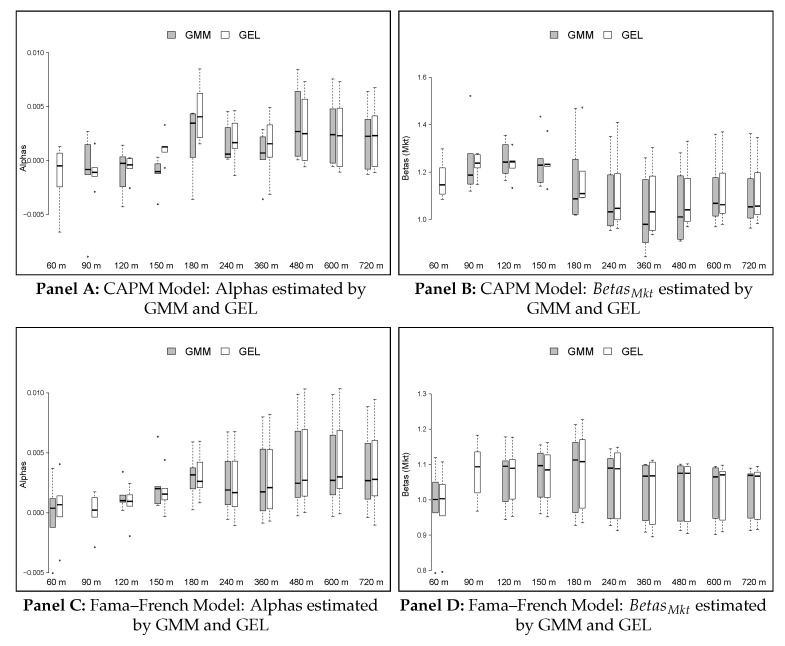
Box plots for comparison of estimations by the GMM and GEL using 6 portfolios based on size and book-to-market for *managed portfolios* for selected periods of time. In all 4 panels, the *x*-axis represents the time intervals, starting from 120 months before December 2014 to 720 months (60 years) prior to this date. The *y*-axis is the estimated coefficient values by the GMM and GEL. The estimations of the GMM are represented by gray box plots, whereas the GEL estimations are represented by white box plots. For T=60 (CAPM) and T=90 (Fama–French), we were not able to estimate the coefficients using the GMM.

**Figure A4:**
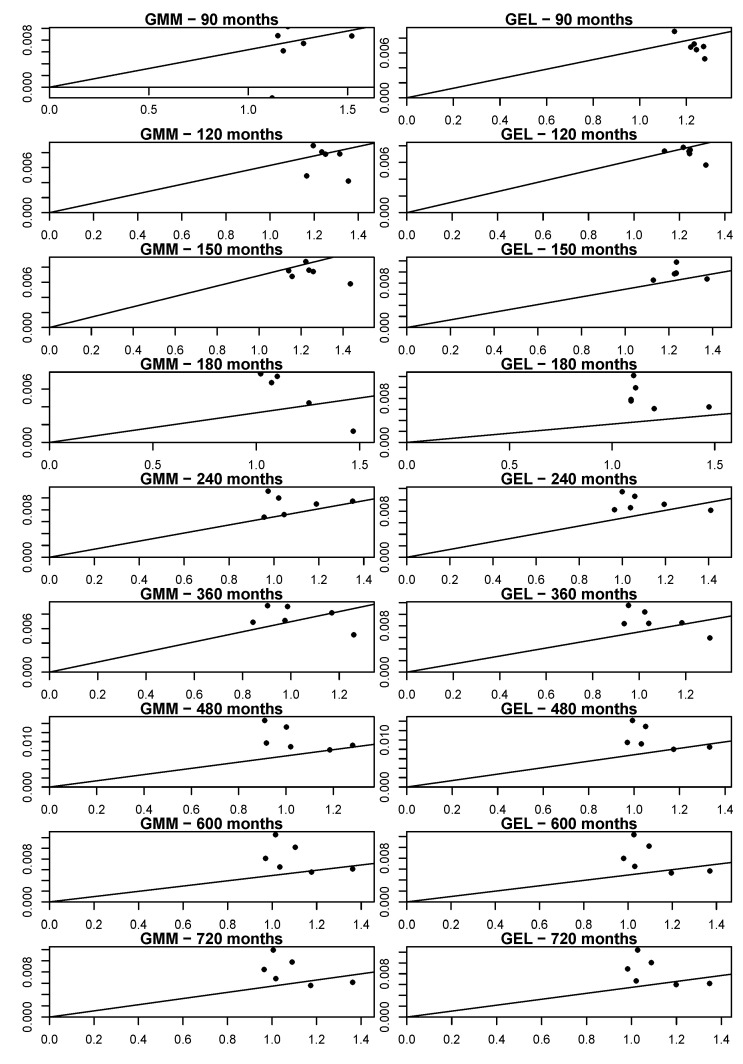
Comparison of the GMM and GEL estimated betas (CAPM) for *managed portfolios* against the sample means of monthly excess returns for the portfolio with 6 assets. In all panels, the estimated betas (${\hat{\beta }}_{Mkt}$) are in the *x*-axis and the sample mean of the monthly excess returns for each of the n=6 assets in the portfolio are in the *y*-axis. Estimations using the GMM are on the left, whereas those using GEL are on the right. Each panel represents one of the time intervals, starting from 120 months before December 2014 to 720 months (60 years) prior to this date.

## Data Availability

All empirical data is from the Kenneth R. French website (http://mba.tuck.dartmouth.edu/pages/faculty/ken.french/data_library.html accessed on 11 November 2022).

## Appendix A. Results for Different Types and Sizes of Portfolios

As discussed in this paper, here, we used different types and sizes of portfolios to evaluate how efficiency tests using either GEL or the GMM can lead to different inference conclusions. Again, we made use of (i) fixed-weight portfolios (no conditional information) and (ii) *managed portfolios* under the *unconditional mean variance efficiency* with respect to the information approach. We considered the CAPM and the Fama–French three-factor models and different sample sizes.

### Appendix A.1. Data—Portfolios with 25 and 49 Assets

In order to examine the estimators’ behaviors for different types of portfolios and higher numbers of assets, we selected two other portfolios. To avoid using a single portfolio composition methodology, the first one was based on size and book-to-market, whereas the second one was composed of categories derived from industry classifications according to the business segment. The data for these portfolios were extracted from the Kenneth R. French website. The chosen portfolios were (i) 25 assets selected with equal weights by size and book-to-market (*25 Portfolios Formed on Size and Book-to-Market (5 × 5)*) and (ii) 49 industry portfolios.

Figure A1 presents the main descriptive statistics of the 25 portfolios based on size and book-to-market. Figure A2 shows the descriptive statistics of the 49 industry portfolios. Notice that both the mean and standard deviations are similar to the previous portfolio, whereas the maximum and minimum returns are magnified. Most of the first-order autocorrelations are lower than 20% and there is only one asset with a negative value. The $R^{2}$ maintains low values, with adjusted coefficients of determination no higher than 5%.

### Appendix A.2. The 25 Portfolios Based on Size and Book-to-Market

#### Appendix A.2.1. No Conditional Information

Table A1 presents the results of the estimations by the GMM and GEL for the portfolio with 25 assets for an increasing sequence of months. Comparing the GMM and GEL for the CAPM, both estimators provide compelling evidence to reject the market portfolio efficiency hypothesis. The *p*-values for both approaches are practically zero for every *T*, except for 120 months using GEL. If we compare these results with other studies regarding the efficiency of a market index, it can be seen that many of them come to similar conclusions [1,47]. Even though the portfolios and the exact time intervals used in these works have a strong resemblance, one should be cautious since they are not exactly the same. However, notice that GEL consistently generated lower test statistics than the GMM (Wald and GRS).

For the Fama–French model, the conclusion to reject the null hypothesis did not change. Here, we had a single case (120 months and GMM) where we were not able to invert the covariance matrix of the alphas. Again, in general, GEL generated lower statistics for both tests (except for 240 months and Fama–French model). This difference seemed to become more relevant as the sample period increased, i.e., we had evidence of an increasing difference relationship when the sample expanded.

**Table A1:** Tests of portfolio efficiency using 25 portfolios based on size and book-to-market for selected periods of time.

	Months	Wald Test			GRS Test			Wald Test			GRS Test	
		Statistic	*p*-Value		Statistic	*p*-Value		Statistic	*p*-Value		Statistic	*p*-Value
		**CAPM**						**FF**				
***GMM***	120	98.5	0.000		3.1	0.000		NA	NA		NA	NA
	240	93.0	0.000		3.3	0.000		114.9	0.000		4.1	0.000
	360	168.7	0.000		6.3	0.000		257.5	0.000		9.5	0.000
	480	123.4	0.000		4.7	0.000		335.7	0.000		12.6	0.000
	600	133.4	0.000		5.1	0.000		465.2	0.000		17.7	0.000
	720	125.5	0.000		4.8	0.000		459.7	0.000		17.7	0.000
	840	122.5	0.000		4.8	0.000		413.2	0.000		16.0	0.000
	960	131.9	0.000		5.1	0.000		398.2	0.000		15.5	0.000
	1020	129.2	0.000		5.0	0.000		403.8	0.000		15.7	0.000
***GEL***	120	53.6	0.001		1.7	0.039		92.1	0.000		2.8	0.000
	240	85.4	0.000		3.0	0.000		138.9	0.000		4.9	0.000
	360	135.0	0.000		5.0	0.000		253.8	0.000		9.4	0.000
	480	90.6	0.000		3.4	0.000		329.0	0.000		12.4	0.000
	600	85.1	0.000		3.3	0.000		362.4	0.000		13.8	0.000
	720	83.2	0.000		3.2	0.000		374.1	0.000		14.4	0.000
	840	80.6	0.000		3.1	0.000		300.4	0.000		11.6	0.000
	960	90.3	0.000		3.5	0.000		300.5	0.000		11.7	0.000
	1020	84.1	0.000		3.3	0.000		310.5	0.000		12.1	0.000

Tests of portfolio efficiency using 25 portfolios based on size and book-to-market (5 × 5) for 9 selected periods of time: T = 120 (10 years), T = 240 (20 years), T = 360 (30 years), T = 480 (40 years), T = 600 (50 years), T = 720 (60 years), T = 840 (70 years), T = 960 (80 years), and T = 1020 (85 years). The tests are conducted based on both estimations’ methodologies, with the GMMresults on the top and the GEL results on the bottom. Tests of efficiency under the CAPM are on the left, whereas tests under the Fama–French three-factor model (represented as “FF”) are on the right. This table presents the statistics and *p*-values of the Wald and GRS tests for each case. “NA” represents situations in which singularity problems occurred, impeding the inversion of the covariance matrix. For *T* = 120 and the Fama–French three-factor model, even though we obtained estimates for the coefficients using the GMM, we were not able to invert the covariance matrix and perform the tests.

#### Appendix A.2.2. Managed Portfolios

Table A2 presents the results of the efficiency tests using the multiplicative approach with five instruments. A quick inspection shows that in many cases, it was not possible to compute the tests Inverting the covariance matrix can become an impediment to the estimation of the tests given the fact that singular matrices can arise. However, there were cases in which we could not estimate the parameters of the model. This situation is represented by “NA” in the table. All “NA” occurred due to the impossibility of the methods to estimate the parameters of the model. Having 25 assets and 5 instruments can cause the optimal long-run covariance matrix to be singular. Singularity problems are a common issue, especially for portfolios with high numbers of assets and under the multiplicative approach with instruments. Ferson and Siegel [1] also had to deal with this issue (see e.g., [48] for advanced treatment).

Note that only for T=480, 600, and 720 was it possible to perform the tests using the GMM. Both tests generated considerably higher estimates (especially Wald) than the fixed-weight approach, leading to very strong evidence for rejecting the null hypothesis. Using GEL, we were not able to estimate the coefficients for any *T*.

**Table A2:** Tests of portfolio efficiency using 25 portfolios based on size and book-to-market for *managed portfolios* for selected periods of time.

	Months	Wald Test			GRS Test			Wald Test			GRS Test	
		Statistic	*p*-Value		Statistic	*p*-Value		Statistic	*p*-Value		Statistic	*p*-Value
		**CAPM**						**FF**				
***GMM***	120	NA	NA		NA	NA		NA	NA		NA	NA
	240	NA	NA		NA	NA		NA	NA		NA	NA
	360	NA	NA		NA	NA		NA	NA		NA	NA
	480	802.7	0.000		30.4	0.000		849.3	0.000		32.0	0.000
	600	480.7	0.000		18.4	0.000		967.6	0.000		36.9	0.000
	720	320.5	0.000		12.4	0.000		851.1	0.000		32.7	0.000
***GEL***	120	NA	NA		NA	NA		NA	NA		NA	NA
	240	NA	NA		NA	NA		NA	NA		NA	NA
	360	NA	NA		NA	NA		NA	NA		NA	NA
	480	NA	NA		NA	NA		NA	NA		NA	NA
	600	NA	NA		NA	NA		NA	NA		NA	NA
	720	NA	NA		NA	NA		NA	NA		NA	NA

Tests of portfolio efficiency using 25 portfolios based on size and book-to-market (5 × 5) for 6 selected periods of time: T = 120 (10 years), T = 240 (20 years), T = 360 (30 years), T = 480 (40 years), T = 600 (50 years), and T = 720 (60 years). The tests are evaluated using conditioning information when instruments are incorporated into the pricing equation. The lagged variables consisting of the conditioning information are the (i) 3-month Treasury-bill yield, (ii) industrial production growth, (iii) yield spreads of low-grade over high-grade corporate bonds, (iv) yield spreads of long-term over short-term Treasury bills (10 year/1 year), and (v) U.S. inflation (CPI). The tests are conducted based on both estimations’ methodologies, with the GMM results on the top and the GEL results on the bottom. Tests of efficiency under the CAPM are on the left, whereas tests under the Fama–French three-factor model (represented as “FF”) are on the right. This table presents the statistics and *p*-values of the Wald and GRS tests for each case. “NA” denotes not applicable for situations in which singularity problems occurred, impeding the inversion of the covariance matrix.

### Appendix A.3. The 49 Industry Portfolios

#### No Conditional Information

Table A3 shows the results of the estimations by the GMM and GEL for the portfolio formed using 49 industrial categories. With this number of assets, singularity issues are more common. Thus, for many sample sizes, we could not perform the tests. Not surprisingly, we were not able to perform the tests of efficiency by either estimation method when using the multiplicative approach (*managed portfolios*).

Examining the results for the periods in which we obtained results, we can see that tests using GEL and the GMM generated high *p*-values (CAPM) and provided no evidence for rejecting the efficiency hypothesis of the market proxy. Note that for these cases, the *p*-values decreased as the sample size increased. For the Fama–French model, although GEL did not provide any test statistics, the GMM results were similar to the CAPM but with higher *p*-values. The *p*-values of the Wald test were 0.98 and 0.69 for 360 and 480 months, respectively, whereas the GRS test *p*-values were 0.99 and 0.84 for the same periods. On the other hand, for the CAPM, the tests of efficiency using GEL had small *p*-values for T=600, whereas the GMM had singularity issues for the same intervals.

These results show that the Wald test, which is based on a large sample distribution, tended to reject the null hypothesis more often than tests that relied on finite sample distributions (such as the GRS). This characteristic is in line with the analysis of the size and power of the efficiency tests by Campbell et al. [49].

**Table A3:** Tests of portfolio efficiency using 49 industry portfolios for selected periods of time.

	Months	Wald Test			GRS Test			Wald Test			GRS Test	
		Statistic	*p*-Value		Statistic	*p*-Value		Statistic	*p*-Value		Statistic	*p*-Value
		**CAPM**						**FF**				
***GMM***	120	NA	NA		NA	NA		NA	NA		NA	NA
	240	41.9	0.753		0.7	0.946		NA	NA		NA	NA
	360	50.6	0.411		0.9	0.685		31.3	0.977		0.5	0.994
	480	60.7	0.123		1.1	0.292		43.7	0.689		0.8	0.838
	600	NA	NA		NA	NA		NA	NA		NA	NA
	720	NA	NA		NA	NA		NA	NA		NA	NA
	840	NA	NA		NA	NA		NA	NA		NA	NA
	960	NA	NA		NA	NA		NA	NA		NA	NA
	1020	NA	NA		NA	NA		NA	NA		NA	NA
***GEL***	120	108.7	0.000		1.3	0.160		NA	NA		NA	NA
	240	48.0	0.514		0.8	0.852		NA	NA		NA	NA
	360	44.1	0.672		0.8	0.860		NA	NA		NA	NA
	480	52.4	0.344		1.0	0.557		NA	NA		NA	NA
	600	81.9	0.002		1.5	0.014		NA	NA		NA	NA
	720	142.8	0.000		2.7	0.000		NA	NA		NA	NA
	840	206.4	0.000		4.0	0.000		NA	NA		NA	NA
	960	212.8	0.000		4.1	0.000		NA	NA		NA	NA
	1020	231.9	0.000		4.5	0.000		NA	NA		NA	NA

Tests of portfolio efficiency using 49 industry portfolios for 9 selected periods of time: T = 120 (10 years), T = 240 (20 years), T = 360 (30 years), T = 480 (40 years), T = 600 (50 years), T = 720 (60 years), T = 840 (70 years), T = 960 (80 years), and T = 1020 (85 years). The tests are conducted based on both estimations’ methodologies, with the GMM results on the top and the GEL results on the bottom. Tests of efficiency under the CAPM are on the left, whereas tests under the Fama–French three-factor model (represented as “FF”) are on the right. This table presents the statistics and *p*-values of the Wald and GRS tests for each case. “NA” denotes not applicable for situations in which singularity problems occurred, impeding the inversion of the covariance matrix.

### Appendix A.4. Additional Results—Portfolio with N = 6

The portfolio evaluated in the paper consists of the six portfolios selected with equal weights by size and book-to-market (*6 Portfolios Formed on Size and Book-to-Market (2 × 3)*). Figure A3 summarizes the estimated coefficients of α and $\beta_{Mkt}$ with the use of instruments for the CAPM and the Fama–French model. As for every *T* and model there are six estimated alphas and betas (Mkt), we present the estimated coefficients for each methodology (GMM and GEL) using box plots. Notice that the box plot distributions are not identical. The GMM and GEL generated different coefficient estimates, not only different covariance matrices, from these estimates. For the CAPM, which is located in the top two panels, notice that for shorter periods, differences are evident between both methodologies. Regarding the $\hat{\alpha }$, the biggest differences are for T=90, 120, 240, and 360. For ${\hat{\beta }}_{Mkt}$, this is clear for sample sizes smaller than 240 months. For the Fama–French model (not shown), differences between the estimates by the GMM and GEL were more subtle. However, for the $\hat{\alpha }$ for very short samples such as 60 months, differences in the estimates were not negligible.

From asset pricing theory, we know that the returns of any asset should be higher if the asset has higher betas. Figure A4 plots the estimated betas (${\hat{\beta }}_{Mkt}$) against the sample means of the monthly excess returns ($\hat{E}\left(R_{i}\right)$) of each of the six assets for the CAPM. The model states that the average returns should be proportional to the betas. Each panel shows one of the time periods evaluated. The GMM estimates are located on the left, whereas the GEL estimates are on the right.

The distances between the points and the straight lines represent the pricing errors, i.e., the estimated alphas. We see a clear difference in the point estimations depending on whether the GMM or GEL was used, with divergence for a short *T* being more evident. Note that in the panels with a *T* less than 180 months, although the estimates using the GMM are more dispersed, those with GEL are more grouped and closer with a slope line equal to $\hat{E}\left(R_{Mkt}\right)$.
